# Editorial: Imaging of Neuromuscular Diseases

**DOI:** 10.3389/fneur.2021.814579

**Published:** 2021-12-02

**Authors:** Jordi Diaz-Manera, Anna Pichiecchio, Francesco Santini, Massimiliano Filosto

**Affiliations:** ^1^John Walton Muscular Dystrophy Research Centre, University of Newcastle, Newcastle upon Tyne, United Kingdom; ^2^Istituto di Ricovero e Cura a Carattere Scientifico Mondino Foundation, Pavia, Italy; ^3^Brain and Behavioral Sciences Department, University of Pavia, Pavia, Italy; ^4^Basel Muscle Magnetic Resonance Imaging (MRI), Department of Biomedical Engineering, University of Basel, Basel, Switzerland; ^5^Division of Radiological Physics, Department of Radiology, University Hospital Basel, Basel, Switzerland; ^6^Department of Research and Analytic Services, University Hospital Basel, Basel, Switzerland; ^7^Department of Clinical and Experimental Sciences, University of Brescia, Brescia, Italy; ^8^NeMO-Neuromuscular OminCenter-Brescia Clinical Center for Neuromuscular Diseases, Brescia, Italy

**Keywords:** neuromuscular diseases, myopathies, neuropathies, imaging, ultrasound, MRI

The heterogeneity of clinical presentation and the rarity of many neuromuscular diseases make it often difficult to achieve the correct diagnosis which is therefore delayed over time.

Recent years have seen a renewed interest in neuromuscular diseases especially due to the emerging innovative therapies, such as molecular therapies, gene therapies, enzyme therapies, already available for some of them such as Duchenne Muscular Dystrophy, Spinal Muscular Atrophy, transthyretin amyloid neuropathy and Pompe disease.

New and exciting therapeutic frontiers await us in the near future and, consequently, early diagnosis has become absolutely necessary for treating patients in the early stages of the disease.

Diagnosis of neuromuscular diseases is historically carried out on the basis of the clinical picture, electromyographic and electroneurographic examination and pathological study by means of a muscle or nerve biopsy.

In recent years, technological development has made available innovative imaging methods that have made it possible to study muscle structures in detail, analyze the severity and distribution of damage and identify specific patterns of damage distribution.

Similarly, ultrasound and MR have become essential tools to support the clinician in diagnosing both peripheral mono- and polyneuropathies (Neurography MR) as well as motor neuron pathology (DTI tractography).

The aim of this Topic Issue was to provide novel original findings on the role of imaging in diagnosing neuromuscular diseases. Several researchers contributed interesting experience and point of views on this subject that is under continuous development.

This topic issue has collected contributions that can be summarized in four macro areas: development of new methods, applications of imaging to muscle diseases, study of peripheral nerve diseases, and evaluation of muscle damage secondary to central nervous system diseases.

## Novel Methods

In general, neuromuscular diseases are rare diseases. For this reason, a higher level of standardization is required in order to compare results across different patients, who are often examined at different imaging centers. To achieve this, standardized quantification and evaluation approaches as well as innovative acquisition methods have emerged.

As regards to the acquisition, the functional aspect of the skeletal muscle is acquiring a prominent role in MR imaging. Mazzoli et al. reported a diffusion-sensitive sequence based on oscillating-gradient spin echo (OGSE) instead of the more common pulsed-gradient spin echo (PGSE) for the acquisition of the water diffusion tensor during muscle contraction. The authors demonstrated fewer artifacts and more reliable quantitative values due to the inherent robustness of this gradient pattern to motion.

On the post processing/analysis aspect, Santini et al. proposed an efficient method for the extraction of quantitative T2 values from multi-echo spin-echo images by using an efficient implementation based on GPU processing. By including an external fat fraction measurement into the processing, the authors demonstrated that it is possible to reduce the acquisition protocol to clinically acceptable times. The fitting method was released as an open source software, an important step toward reproducibility.

When specialized acquisitions are not available, Akinci D'Antonoli et al. demonstrated the utility of texture analysis and machine learning methods to assist the diagnosis of cerebral palsy (CP) cases. Texture analysis extracts quantitative features from qualitative images (in this case, in-phase gradient-echo images) and it potentially improves objectivity and reader-dependency. In their paper, the authors demonstrated the superiority of their texture analysis approach with respect to simple fat fraction for the diagnosis of paretic muscles in CP.

All quantitative evaluations of the imaging features in neuromuscular diseases, however, depend on an accurate and reliable segmentation of the muscle groups. This is a challenging task because of deformable geometry, different appearance due to pathological processes, and the limited number of cases available for each site. Ogier et al. gave a detailed overview of the state-of-the-art methods for muscle segmentation including different algorithms and strategies. Atlas-based and conventional image analysis methods are currently being replaced by deep-learning-based methods. While these methods are showing promising results, the availability of large amounts of labeled data is a limit that needs to be addressed for the building of accurate models.

## Imaging Applied to the Study of Muscle Disorders

Muscle MR has already demonstrated to be an useful tool for the diagnosis and follow-up of patients with a wide variety of primary muscle disorders including both acquired and inherited myopathies. There are a large number of studies using conventional MR sequences such as T1-weighted and STIR describing the changes in muscle structure of patients at different stages of disease progression. T1w imaging is useful as it identifies fat tissue in the skeletal muscles and allows to describe the so-called patterns of muscle involvement which could guide the diagnosis. But as T1w allows to quantify fat replacement, these data have been successfully used to study correlation with results of muscle function.

Here, Reyes-Leiva et al. described the correlation between the amount of fat replacement in respiratory accessory muscles and the results of the spirometry in a large cohort of patients with late onset Pompe disease, confirming that fat replacement of these muscles could be used as a biomarker of disease progression. In this sense quantification of fat through muscle MR can also be used to classify patients as severely or moderately affected leading to the identification of risk factors that can be associated to one or the other group.

Moore et al. studied a large cohort of patients with dysferlinopathy and identified that performing exercise during adolescence was associated with a more severe fat replacement on the pelvis, thigh and leg muscles and to worse results on muscle function tests. Quantification of muscle fat replacement by means of Dixon sequences has proven to be more powerful to identify changes over time in muscle fat content than semi quantitative methods. Moreover, in most of these studies, muscle function tests are stable over short periods of time although fat content increases as detected by quantitative sequences, suggesting that subtle changes in muscle structure, which are a sign of disease progression, are not always translated into changes in muscle function.

Sheikh et al. included 16 Becker muscular dystrophy patients in 1 year longitudinal study where axial and lower limb muscles were imaged using Dixon MRI and their strength was assessed using Biodex. The authors observed that frat fraction was higher in paraspinal muscles in Becker patients compared to controls and that there was an inverse significant correlation between the fat fraction and muscle strength of the paraspinal muscles. Moreover, they also observed significant differences in fat replacement over 1 year period of time in the paraspinal muscles using Dixon in those patients that were less affected at baseline while there were no differences in their muscle strength suggesting that MRI can be a stronger biomarker for disease progression than muscle function test in clinical trials or natural history studies.

Detection of fibrosis is one of the unmet needs of muscle MRI. Although there have been several attempts to identify fibrotic tissue, so far there is not any reliable MRI sequence able to identify and/or quantify collagen in the skeletal muscles. Murphy et al. successfully used EP3533, which is a novel MRI contrast agent with an affinity to collagen 1 that correlates to *ex vivo* fibrosis quantification, to study response to treatment of mdx mice treated with halofuginone, a well-known anti-fibrotic compound. They were able to observe an increase in fibrotic tissue in the non-treated animals that was statistically higher than the treated group. Histological findings were in agreement with EP35333 imaging. Although further studies in humans are needed, this study opens the door to use contrast agents to detect and quantify fibrotic content in muscles of patients with muscular dystrophies. Other components of the skeletal muscles can also be detected using specific sequences such as glycogen. However, these innovative sequences are generally not available in hospitals.

With the aim to identify indirect changes on muscle MRI conventional sequences of glycogen, Nuñez-Peralta et al. studied a cohort of patients with late onset Pompe disease with magnetization transfer sequence which analyzed the transfer imaging that exploits the interaction between bulk water protons and protons contained in macromolecules. Authors observed a decreased magnetization transfer ratio (MTR) in skeletal muscles of patients with Pompe disease that correlated with fat content measured using Dixon suggesting that MTR is a good marker of loss of muscle fibers and substitution by fatty tissue. Authors however were not able to see any changes in muscles with low levels of fat replacement, suggesting that if glycogen is accumulated in these muscles this is not inducing any change in MTR.

## Imaging Applied to the Study of Peripheral Nervous System

The diagnostic workup of peripheral neuropathies is traditionally based on clinical history, physical examination and electrophysiological studies. In the last few years, novel MR and ultrasound (US) imaging techniques have been developed for studying the peripheral nervous system which have increased the diagnostic rate in this field of neurology.

Ultrasound is very suitable for evaluation of superficial peripheral nerves and MR is especially useful in studying deeply located nerves.

Carpal tunnel syndrome (CTS) is one of the most common upper limb compression neuropathies which is usually diagnosed by clinical history and examination and electrophysiological studies. Ultrasonography is commonly used to confirm diagnosis after electrodiagnostic testing and a median nerve CSA increase at the carpal tunnel inlet is usually observed. Fixed values for the upper limit of normal (ULN) with a broad range of 8.5–15 mm^2^ are reported.

In a first study Dubbelink, De Kleermaeker, Beekman et al. showed that the use of a wrist-circumference dependent cut-off value for the CSA of the median nerve at the wrist has a higher sensitivity than using a fixed cut-off of 11 mm^2^ or an intraneural flow related cut-off and may be especially useful in patients with a smaller wrist circumference.

In a second study, Dubbelink, De Kleermaeker, Meulstee et al. measured CSA and wrist circumference in a prospective cohort of 253 clinically defined CTS patients and 96 healthy controls and developed an equation for the ULN for CSA by means of univariable regression analysis. They found an augmented diagnostic accuracy of this newly developed equation with a corresponding sensitivity and specificity of 75% compared to a sensitivity of 70% by using a fixed cut-off value of 11 mm^2^ and therefore improving diagnostic accuracy of ultrasonography in patients with CTS.

Nerve ultrasound is useful in differential diagnosis of polyneuropathies.

Du et al. evaluated whether ultrasound is suitable for differentiating Transthyretin familial amyloid polyneuropathy (TTR-FAP) and chronic inflammatory demyelinating polyneuropathy (CIDP) because misdiagnosis is frequent as of similar phenotypes. By performing consecutive ultrasonography scanning in six pairs of nerves of bilateral limbs with 30 site and comparing CSAs and CSA variability data in 18 patients with TTR-FAP, 13 patients with CIDP and 14 healthy controls, they showed that both TTR-FAP and CIDP present larger CSAs at most sites of both upper and lower limbs than in control groups but the CSA variability of median nerves is significantly higher in CIDP than in TTR-FAP and control groups with high sensitivity and specificity to differentiate CIDP from TTR-FAP, therefore suggesting that nerve ultrasound can be a potential auxiliary tool to help differentiate the two polyneuropathies.

Imaging can also be a very useful tool in detecting involvement of peripheral nervous system when clinical and electrophysiological assessments are inconclusive.

Kim and Sung studied by MR two cases of neurogenic thoracic outlet syndrome (N-TOS) which is a chronic compressive brachial plexopathy involving the C8 and T1 roots, and/or lower trunk. Although patients showed typical neurological symptoms of N-TOS and structural abnormalities of the thoracic outlet (cervical rib and anomalous first rib), no neurological deficit at the neurologic examination and no abnormalities in the electrophysiological studies were observed. Diagnosis was achieved by CT angiography showing abnormalities of the subclavian artery and root-plexus MR revealing anomalies of nerve root or lower trunk.

Mandelli et al. studied paraspinal muscles by MR in patients with symptomatic Lumbar spinal stenosis in order to compare paraspinal muscle fatty infiltration as assessed using the Goutallier classification vs. quantitative MR measurements. They found that the Goutallier classification is a reliable tool for assessing fatty infiltration of paraspinal muscles in this group of patients and suggest taking body height as a reference for normalization.

## Cns Diseases

Muscles can also be secondly involved in different disorders such as cerebral palsy (CP) or spinal muscular atrophy (SMA).

Muscles from patients with CP are usually spastic and present contractures that limit the range of motion. Weidensteiner et al. assessed by MR the effect of botulinum toxin A (BTX) over time on calf muscle properties in pediatric CP patients. BTX induced increase in extracellular space and a simultaneous decrease of muscle fiber diameter and MRI showed limited spatial distribution of these BTX-induced effects, being a promising non-invasive tool for future studies in order to test BTX treatment protocols.

On the other hand, in muscles of patients affected by SMA3 treated with nusinersen Savini et al. found a progression of fat fraction (FF) in thigh muscles and a concurrent slight reduction of water T2 over time, despite therapy. Current advances in morphometric MRI development also allow gray (GM) and white matter (WM) quantification in the spinal cord (SC), which may help in improving the *in vivo* characterization of neurodegenerative SC diseases or lower motor neuron disorders such as SMA. These advanced MR techniques have been applied together with muscle quantitative MR in the pilot study of Savini et al. on three adult SMA3 patients under treatment with nusinersen to characterize SC volumes and microstructure (GM and ventral horns) as well. The authors demonstrated that quantitative cervical SC MR sequences derived from the consensus acquisition protocol produced by a consortium of SC researchers (1) including 3D T1- and T2 weighted (w), DWI, MT and T2^*^-w sequences were able to capture microstructural changes induced by SMA *in vivo* and are a candidate methodology for monitoring the effects of this treatment.

Different SC imaging approaches are also appearing on the horizon, such as radially sampled averaged magnetization inversion recovery acquisition (rAMIRA), which is a novel approach to perform SC imaging in clinical settings with favorable contrast. These approaches need to be tested on healthy subjects (HS) to understand the sources of inter-subject variability in different SC levels as well as their relation to age and sex to facilitate the detection of pathology-associated changes. Kesenheimer et al. identified effective normalization strategies for inter-subject variability reduction in total SC area and SC GM area on a broad aged cohort of HS using rAMIRA.

Taken together, the papers collected in this Issue present several interesting and original findings which may contribute to the knowledge on the role of imaging in neuromuscular disorders and offer novel perspectives for future studies and clinical applications.

## Author Contributions

All authors listed have made a substantial, direct, and intellectual contribution to the work and approved it for publication.

## Conflict of Interest

The authors declare that the research was conducted in the absence of any commercial or financial relationships that could be construed as a potential conflict of interest.

## Publisher's Note

All claims expressed in this article are solely those of the authors and do not necessarily represent those of their affiliated organizations, or those of the publisher, the editors and the reviewers. Any product that may be evaluated in this article, or claim that may be made by its manufacturer, is not guaranteed or endorsed by the publisher.
